# Qingkailing injection ameliorates cerebral ischemia-reperfusion injury and modulates the AMPK/NLRP3 Inflammasome Signalling pathway

**DOI:** 10.1186/s12906-019-2703-5

**Published:** 2019-11-20

**Authors:** Chongyang Ma, Xueqian Wang, Tian Xu, Xue Yu, Shuang Zhang, Shuling Liu, Yushan Gao, Shuning Fan, Changxiang Li, Changming Zhai, Fafeng Cheng, Qingguo Wang

**Affiliations:** 1 School of Traditional Chinese Medicine, Capital Medical University, 10 Youanmenwai, Xitoutiao, Fengtai District, Beijing, 100069 China; 2 School of Traditional Chinese Medicine Department, Beijing University of Chinese Medicine, 11 Beisanhuandong Road, Chao Yang District, Beijing, 100029 China

**Keywords:** Cerebral ischemia-reperfusion injury, NLRP3 inflammasome, Chinese medicine, Qingkailing injection

## Abstract

**Background:**

Cerebral ischemia is the second-leading cause of death and the main cause of permanent adult disabilities worldwide. Qingkailing (QKL) injection, a patented Chinese medicine approved by the China Food and Drug Administration, has been widely used in clinical practice to treat cerebral ischemia in China. The NOD-like receptor pyrin 3 (NLRP3) inflammasome is activated in cerebral ischemia and thus, is an effective therapeutic target. AMP-activated protein kinase (AMPK) is an important regulator inhibiting NLRP3 inflammasome activation.

**Methods:**

We investigated the potential of QKL injection to provide neuroprotection after cerebral ischemia in a rat model of middle cerebral artery occlusion (MCAO). Adult male Sprague-Dawley rats (210–230 g) were randomly divided into three groups which consist of sham, MCAO and 3 ml/kg QKL. Rats in the QKL group received intraperitoneal injections of 3 ml/kg QKL, while rats in other groups were given saline in the same volumes. After 90 min ischemia and 24 h reperfusion, neurological function, laser speckle imaging, brain infarction, brain water content and brain blood barrier permeability were examined and cell apoptosis at prefrontal cortex were evaluated 24 h after MCAO, and western blot and real-time quantitative polymerase chain reaction was also researched, respectively.

**Results:**

Intraperitoneal administration of QKL alleviated neurological deficiencies, cerebral infarction, blood-brain barrier permeability, brain oedema and brain cell apoptosis after MCAO induction. QKL decreased pro-inflammatory cytokines, TNF-α, IL-6 and IL-1β, and increased anti-inflammatory cytokines, IL-4 and IL-10. Furthermore, QKL activated phosphorylated AMPK, decreased oxidative stress and decreased NLRP3 inflammasome activation.

**Conclusions:**

QKL relieved cerebral ischemia reperfusion injury and suppressed the inflammatory response by inhibiting AMPK-mediated activation of the NLRP3 inflammasome. These results suggest that QKL might have potential in treating brain inflammatory response and attenuating the cerebral ischemia-reperfusion injury.

## Background

Cerebral Ischemia is the second-leading cause of death behind ischemic heart disease, and is the main cause of permanent adult disabilities worldwide [1, 2]. Thrombolytic therapy is the only therapy recommended to treat cerebral ischemia, however, it is limited by a very narrow therapeutic window and a high risk of haemorrhagic complications [3]. Therefore, a focus on understanding the detailed pathological process behind cerebral ischemia may facilitate the creation of more novel and efficient therapeutic agents. The importance of innate immune mechanisms as a response to cerebral ischemia-reperfusion injury has been recognized recently [4]. Following a transient blockage of cerebral blood flow, dangerous molecular signals are released from dead or dying cells [5]. These signals, known as damage-associated molecular patterns (DAMPs) and pathogen-associated molecular patterns (PAMPs), stimulate the initial activation of innate immune responses during the progression of cerebral ischemia via extracellular and intracellular pattern recognition receptors (PRRs). Inflammasomes are activated by some PRR signals, induce maturation and secretion of some inflammatory cytokines, and initiate cell pyroptosis, a form of programmed inflammatory cell death [6].

Recent research has highlighted a novel inflammasome, the nucleotide-binding oligomerization domain (NOD)-like receptor (NLR) Pyrin domain containing 3 (NLRP3) inflammasome that acts as a key regulator in detecting cellular damage and modulating inflammatory responses to aseptic tissue injury post-stroke [7]. NLRP3 inflammasome is one of the best characterized inflammasomes to date, and is the most strongly relevant in cerebral ischemia. The NLRP3 inflammasome comprises three kinds of cytoplasmic proteins: 1) NLRP3, 2) apoptosis-associated speck-like protein containing a CARD (ASC), and 3) a precursor of caspase-1, which cleaved formation leads to maturation and secretion of IL-1β and IL18, and induce cellular pyroptosis [8]. In the central nervous system (CNS), an NLRP3 inflammasome signalling pathway was activated and the expression of core proteins, such as NLRP3, ASC, caspase-1, IL-1, and IL-18, were upregulated in vitro and in vivo under ischemic conditions [9]. Suppressing the NLRP3 inflammasome activation was also proven to be associated with better functional outcomes, decreased infarction volumes and oedema formation, preserved blood brain barrier (BBB) permeability, and reduced inflammatory pathology in a transient middle cerebral artery occlusion (tMCAO) rat model [10, 11].

AMP-activated protein kinase (AMPK) is a master sensor of cellular energy balance and a fundamental regulator of cellular carbohydrate and fat metabolism and ATP conservation and synthesis. An increased AMP: ATP ratio leads to AMPK kinase activation and then activates AMPK to switch off ATP-consuming pathways and switch on ATP-generating pathways [12]. Recently, AMPK was found to play a role in regulating NLRP3 inflammasome activation. According to a newly published review article, activating AMPK signals leads to inhibition of the NLRP3 inflammasome via improved autophagy, alleviation of ER stress, activation of SIRT1, and regulation of mitochondrial homeostasis [13]. AMPK activation represents a potential protective mechanism in the early stages of cerebral ischemia [14]. Therefore, the AMPK/NLRP3 inflammasome pathway has the potential to be a therapeutic target in the treatment of cerebral ischemia.

Qingkailing (QKL) injection, a patented Chinese medicine that is approved by the China Food and Drug Administration to treat cerebral ischemia (registration information can be accessed here: http://samr.cfda.gov.cn/WS01/CL0412/), was originally prepared by a group of scientists at the Beijing University of Chinese Medicine in the 1970s. The substance was formulated by modifying a famous traditional Chinese medicine, Angongniuhuang [15], which were pills composed of Radix Isatidis, Flos Lonicerae, Concha Margaritifera Usta, baicalin, Fructus gardeniae, cholic acid, hyodeoxycholic acid, and Cornu Bubaliand. Angongniuhuang pills had been widely used in clinical practice to treat cerebral ischemia for over 30 years. A recently updated systematic review and meta-analysis published by our group evaluated 18 randomized controlled trials (RCTs, 1722 patients). The results indicated that QKL Injection combined with a conventional therapy improved the efficiency of treatment for cerebral ischemia compared to conventional treatment alone [16, 17]. The specific mechanism behind the biological effect of QKL at that time, however, was not fully understood. Recent real world study showed that QKL decreased the level of C-reactive protein, white blood cells and other abnormal inflammatory indicators clinically, highlighting a pharmacological effect on immune system [18]. And our unpublished systems pharmacology results showed that QKL may regulate post-stroke inflammatory response and modulate pattern recognition receptors signal. In vitro evidence showed that QKL significantly supressed the activation of microglia and modulated secretion of inflammatory factors, including TNF-α, COX-2 and iNOS [19].

In the present study, we hypothesized that QKL could ameliorate cerebral ischemia-reperfusion injury and modulate the AMPK/NLRP3 inflammasome signalling pathway. To test our hypothesis, we designed an experiment that could quantify the brain infarct volume and blood-brain barrier permeability and detect the level of activation of the AMPK/NLRP3 pathway.

## Methods

### Ethical statement

The animal experimental design and protocols used in this study were approved by the Ethics Review Committee for Animal Experimentation at the Beijing University of Chinese Medicine. All the experimental procedures were performed in accordance with the Regulations for the Administration of Affairs Concerning Experimental Animals approved by the State Council of People’s Republic of China.

### Drugs and reagents

Qingkailing (QKL) was purchased from Shenwei Pharmaceutical Co., Ltd. (No. Z13020935, China). The protease inhibitor, radioimmunoprecipitation assay (RIPA) lysis buffer, and enhanced chemiluminescence (ECL) reagent were obtained from Vazyme Biotech (Nanjing, China). The antibodies against GAPDH, p-AMPK, and AMPK were purchased from Cell Signaling Technology (Danvers, MA USA), and the antibodies against NLRP3, ASC, IL-1β and caspase-1 were obtained from Abcam (Cambridge, UK).

### Animals

Adult male Sprague-Dawley rats (210–230 g) were provided by the Vital River Laboratory Animal Technology (number SCXK 2016–0006) (Beijing, China). The rats were housed in the experimental animal centre of Beijing University of Chinese Medicine, which was maintained at 25 °C ± 1 °C with 65% ± 5% humidity on a 12-h light/dark cycle for at least 1 week before the experiments. Animals were given food and water freely.

### Model establishment of tMCAO and treatment

All animals were fasted overnight but allowed free access to water and were assigned to three groups according to the random number table (*n* = 22 rats each group) as follows: (1) Sham group, (2) MCAO group and (3) QKL group. The transient MCAO model was performed as described previously [20]. Briefly, rats were anesthetized 4% isoflurane until no corneal reflex and toe-pinching were observed. And 1% isoflurane in a mixture of 30% oxygen and 70% nitrous oxide was used during tMCAO surgery. A poly-L-lysine-coated nylon suture was inserted from the right external carotid artery into the common carotid artery to occlude the middle cerebral artery. Rats in the sham group were suffered similar operation without inserting sutures. Cerebral reperfusion was carried out by carefully removing the suture after 90 min from the onset of occlusion. The body temperature was controlled at 37 °C by an electric blanket. Rats in the QKL group received intraperitoneal injections of QKL dissolved in saline at a dose of 3 ml/kg. Rats in the MCAO group and Sham group received intraperitoneal injections of saline in the same volumes. The first injection was performed immediately after model establishment, followed by an administration after 4 h, and then once every 12 h thereafter. At 24 h after reperfusion, euthanasia was performed by excessive inhalation of isoflurane. Death was monitored by the cardiac activity and respiration. All efforts were made to minimize animal suffering and to reduce the Number of animals used. All efforts were made to minimize animal suffering and to reduce the Number of animals used. After sacrificed, all the brains were taken out quickly for TTC staining (*n* = 10 each group), assessment of BBB permeability and brain water containing (*n* = 6 each group), histomorphological assays (*n* = 3 each group) or protein detection (n = 3 each group).

### Neurological assessment

The neurological deficit for each rat was measured 24 h after reperfusion by an investigator who did not know the experimental groupings using the a five-point neurological scale [21]: score 0 = no apparent deficits; score 1 = failure to fully extend the right forepaw; score 2 = circling to the right; score 3 = falling or leaning over to the right; score 4 = no spontaneous walking and a depressed level of consciousness; score 5 = dead.

### Cerebral blood flow measurement

After neurological assessment, cerebral blood flow (CBF) was measured by Lasser Doppler perfusion monitor (PeriCam PSI System) as previously described [22, 23]. Rats were anesthetized with the dose of 35 mg/kg pentobarbital sodium solution (3%, w/v). Laser scanning imaging measurements were performed on the intact skull. Real-time CBF changes were recorded every 1 min with a CCD camera and a Pericam PSI System (Perimed) that was placed roughly 10 cm above the brain. From the measurements obtained, the relative ipsilateral: contralateral CBF ratio was calculated.

### Infarct volume assessment

Following the neurological function evaluation and cerebral blood flow measurement, the rats were sacrificed as described previously and the brains were harvested for TTC staining [24, 25]. Brains were cut into serial coronal sections (2 mm thickness) and soaked in 2% TTC phosphate buffer at 37 °C for 15 min in the dark place. These sections were soaked in 4% paraformaldehyde phosphate buffer for 30 min. An electronic scanner (Tsinghua Unisplendour A688, Xi’an, China) was used to capture images of ordered brain sections. Normal brain tissues stained red while infarct tissues did not stain (white). Areas of red and white staining were measured using a computer colour multimedia image analysis system (Image-Pro Plus6.0, Media Cybernetics, Wyoming, USA). The percent of infarction was calculated using the following equation: %Infarct volume = Infarct volume/Total volume of slice × 100.

### Brain water content

Brain oedema was also determined 24 h after reperfusion. The brains were separated and sliced. The slices were immediately weighed and recorded as the wet weight, and then they were put in an oven at 100 °C for over 24 h to obtain the dry weight. The water content was expressed as using following formula: [(wet weight) – (dry weight)]/(wet weight) × 100%.

### BBB permeability

BBB permeability was measured using Evans Blue (EB) extravasation at 24 h after reperfusion [26]. In brief, EB dye (2%, 4 mL/kg) was injected through caudal vein at a dose of 2 ml/kg and allowed to circulate for 1 h. The rats were anesthetized using pentobarbital sodium solution and perfused transcardially using cold saline to remove intravascular EB dye. After sacrificed, the entire brain of each animal was removed, divided into ipsilateral and contralateral hemispheres, and homogenized in physiological phosphate buffered saline (PBS). Trichloroacetic acid was then added to precipitate the proteins, and the samples were cooled and centrifuged. The supernatant was measured for EB absorbance at 620 nm using a spectrophotometer. Taking the optical density of the contralateral hemisphere as background, the fold change was determined by the following formula: (ipsilateral-contralateral)/contralateral.

### Nissl staining

Coronal brain sections were stained with thionine as previously described to assess ischemic/reperfusion injury [27]. Necrotic neurons were identified by the disappearance of Nissl bodies in the cytoplasm, shrunken intercellular spaces, and deep staining. The number of neuronal cells in the border of the infarct area was counted.

### TUNEL staining

Cerebral ischemia-induced apoptosis was quantified by TdT-mediated dUTP Nick-End Labelling (TUNEL) assay (Roche Molecular Biochemicals, Mannheim, Germany) as previously described [28, 29]. TUNEL-positive apoptotic cells in peri-ischemia region of cortex were counted in five random high-power fields per section by an investigator who did not know the experimental groupings. The apoptotic index was calculated as a ratio of the apoptotic cell number to the total cell number in each field.

### ELISA analysis

The cortical tissue in the penumbra was washed in cold PBS and shredded below 4 °C. Then cell lysates were centrifuged at 1000 g for 20 min, and supernatant was stored at − 20 °C. The concentration of TNF-α, IL-6, IL-1β, IL-4 and IL-10 in the cortex was assessed using ELISA, according to the manufacturer’s protocol (Cloud-Clone Corp, Texas, USA). The total protein level was normalized for each sample to conduct ELISA [30].

### MDA and SOD measurement

MDA was determined using a kit according to the manufacturer’s directions (Nanjing Jiancheng Bioengineering Institute). In addition, SOD activity was determined using the xanthine oxidase method according to the manufacturer’s directions (Nanjing Jiancheng Bioengineering Institute).

### Western blotting

The cortical tissue in the penumbra was washed in cold PBS and shredded below 4 °C. The total protein was extracted using RIPA lysis buffer containing protease inhibitor and phosphatase inhibitor, and the concentration was measured using a bicinchoninic acid (BCA) Protein Assay Kit (#CW0014, CWbio, China). Proteins of 50 μg were separated on sodium dodecyl Right (SDS)-polyacrylamide gels and transferred onto a polyvinylidene fluoride membrane (Millipore Corporation, Billerica, USA). The membrane was blocked with 5% nonfat dry milk in Tris-buffered saline containing 0.05% Tween-20 (TBST) buffer. They were then incubated with primary antibodies against NLRP3, ASC, caspase-1, IL-1β, p-AMPK, AMPK, and GAPDH (dilutions of 1:1000, 1:1000, 1:1000, 1:1000, 1:2000, 1:2000, 1:10000) overnight at 4 °C. The membranes were incubated for 1 h at room temperature with secondary antibodies coupled to horseradish peroxidase at a 1: 10000 dilution. The antigen-antibody complexes were then tested by enhanced chemiluminescence (ECL) reagent and visualized on a C600 Western Blot Imaging System (Azure Biosystems, Dublin, USA). The protein levels of these proteins are expressed as relative integrated intensity normalized versus GAPDH.

### Quantitative real-time PCR quantitation

RNA was isolated from the cortical tissue in the penumbra using Trizol Reagent (Invitrogen, Grand Island, NY, USA). The RNA concentration was measured using an ultraviolet spectrophotometer (UV-2000, Unico, Shanghai, China). Then, reverse transcription was performed from 2 μg RNA on a T100 Thermal Cycler PCR machine (Bio-Rad, USA) using Revert Aid First Strand cDNA Synthesis Kit (Thermo Fisher Scientific, Waltham, WA, USA,). Quantitative PCR was performed according to the manufacturers’ instructions using SYBR Select Master MIX (K1622, Applied Biosystems, CA, USA) and a Real-Time PCR machine (Bio-Rad, USA). Expression values of the targeted genes were normalized to the corresponding expression of β-actin. The 2^−ΔΔCt^ method was used to calculate the relative gene expression. Sequence-specific primers are listed in Table 1.

**Table 1:** Real-time fluorescent quantitative PCR primers

Gene names	Forward	Reverse
NLRP3	CAGCGATCAACAGGCGAGAC	AGAGATATCCCAGCAAACCTATCCA
ASC	GCTGAGCAGCTGCAAACGA	ACTTCTGTGACCCTGGCAATGA
Caspase-1	ACTCGTACACGTCTTGCCCTCA	CTGGGCAGGCAGCAAATTC
IL-1β	CCCTGAACTCAACTGTGAAATAGCA	CCCAAGTCAAGGGCTTGGAA
GAPDH	GAACATCATCCCTGCATCCA	CCAGTGAGCTTCCCGTTCA

### Statistical analysis

All experiments were performed in triplicate and the data are expressed as means ± standard error measurements (SEM). One-way analysis of variance (ANOVA) were employed for the statistical analysis using SPSS 22.0 software (SPSS, Inc.). *P* < 0.05 was defined as significant difference.

## Results

### Effects of QKL on regional cerebral blood fluid, neurological score, and infarct volume

**Fig. 1:**
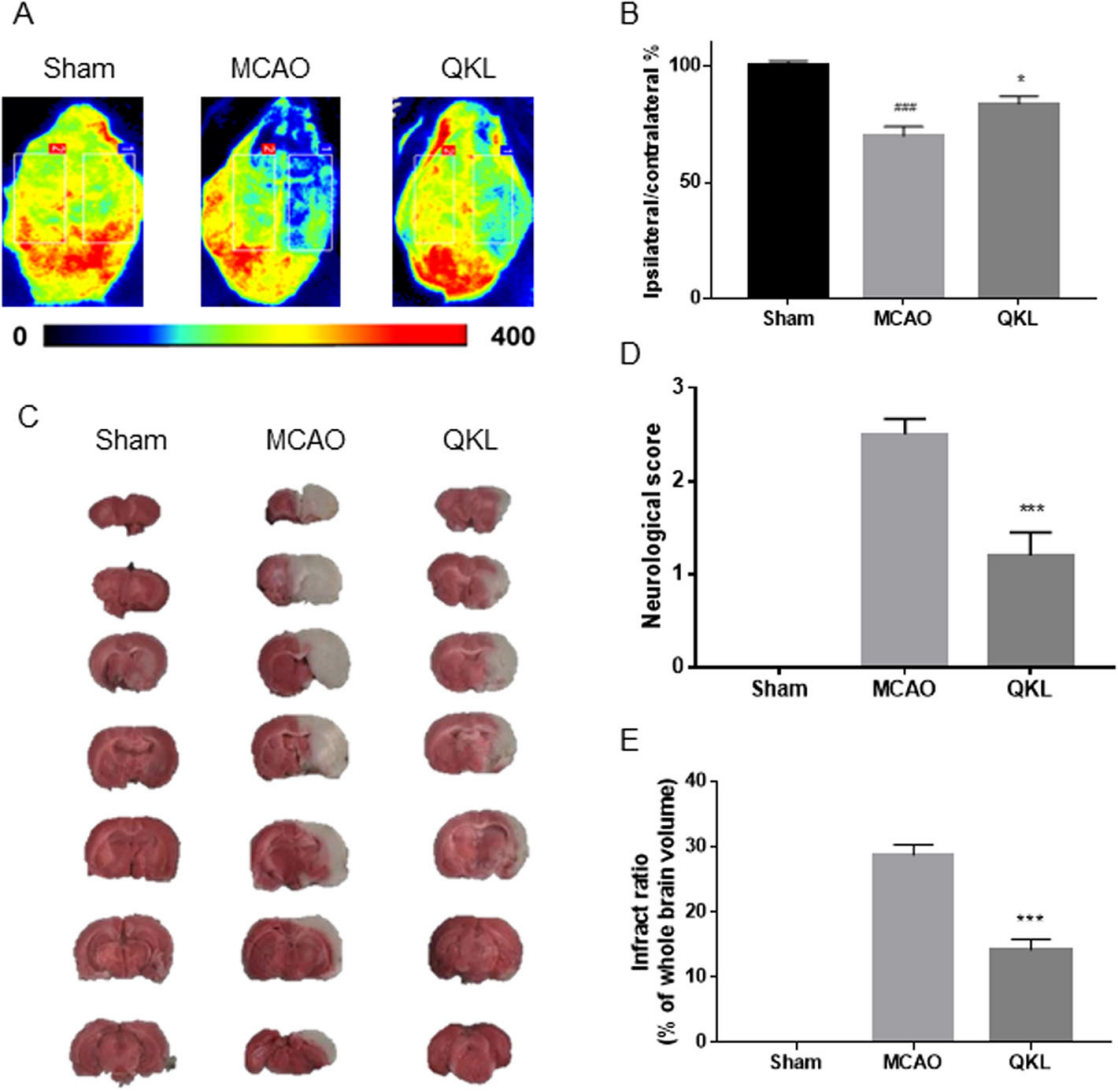
Effects of Qingkailing (QKL) injection on ischemic injury in vivo. **a** Effects of QKL on region cerebral blood flow. **b** Quantitative analysis of regional cerebral blood flow. **c** Representative pictures of brain sections stained with 2% TTC. **d** Effect of QKL on neurological deficit scores. **e** Quantitative analysis of cerebral infarct volume. Data points indicate means ± SEM from 10 individual rats in each group. Vs Sham group, ### < 0.001, Vs MCAO group, * *p* < 0.05, *** *p* < 0.001

To evaluate the hemodynamic effects induced by the QKL injection, we analysed regional CBF in the ipsilateral and contralateral tissues using the PeriCam PSI System (PerimedAB, Sweden). We used the ipsilateral: contralateral rCBF ratio to evaluate the effect of QKL on the decrease in blood flow post-stroke (F (2, 27) = 12.73, *p* = 0.0003). As shown in Fig. 1a-b, 24 h after the cerebral ischemia-reperfusion injury, the rCBF of the ipsilateral side, after being normalized by the contralateral side, decreased to 69.92% (Vs Sham, *p* = 0.0002) and QKL reversed the data to 83.85% (Vs MCAO, *p* = 0.0181). We also observed that QKL improved neurological function and induced brain infarct volume as shown in Fig. 1c-e.

### Effects of QKL on BBB permeability and brain water content

**Fig. 2:**
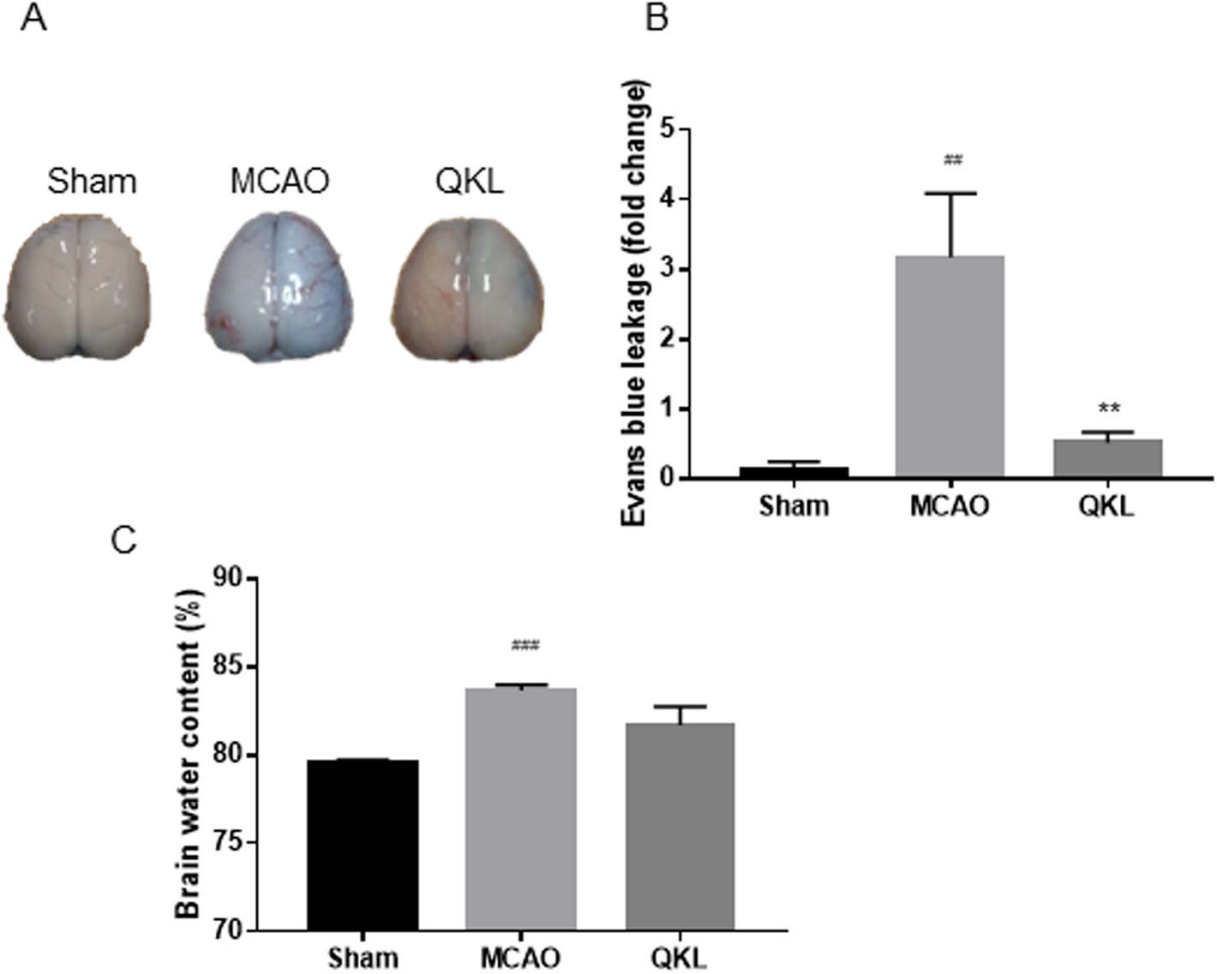
Effects of QKL on post-stroke blood-brain barrier (BBB) dysfunction. **a** Representative pictures of Evans blue (EB) leakage analysis. **b** EB leakage analysis to evaluate prevention of BBB dysfunction by QKL. **c** Brain water content of each hemisphere. Data points indicate means ± SEM from 6 individual rats in each group. Vs Sham group, ## < 0.01, ### < 0.001, Vs MCAO group, ** *p* < 0.01

We evaluated the BBB permeability by assessing Evans blue (EB) extravasation at 24 h post-stroke (F (2, 15) = 6.575, *p* = 0.0023). The EB leakage was significantly reduced in the QKL group than in the MCAO group as shown in Fig. 2a-b (Vs MCAO, *p* = 0.0065). And the cerebral ischemia-reperfusion injury resulting BBB leakage caused an increase in brain water content (Vs Sham, *p* = 0.0024). Although there is a significant difference among these groups (F (2, 15) = 2.166, *p* = 0.0015), as shown in Fig. 2c, QKL administration led to a decrease in brain oedema in the ipsilateral hemisphere, and the difference was not significant (Vs MCAO, *p* > 0.05).

### Effects of QKL on post-stroke cell survival

**Fig. 3:**
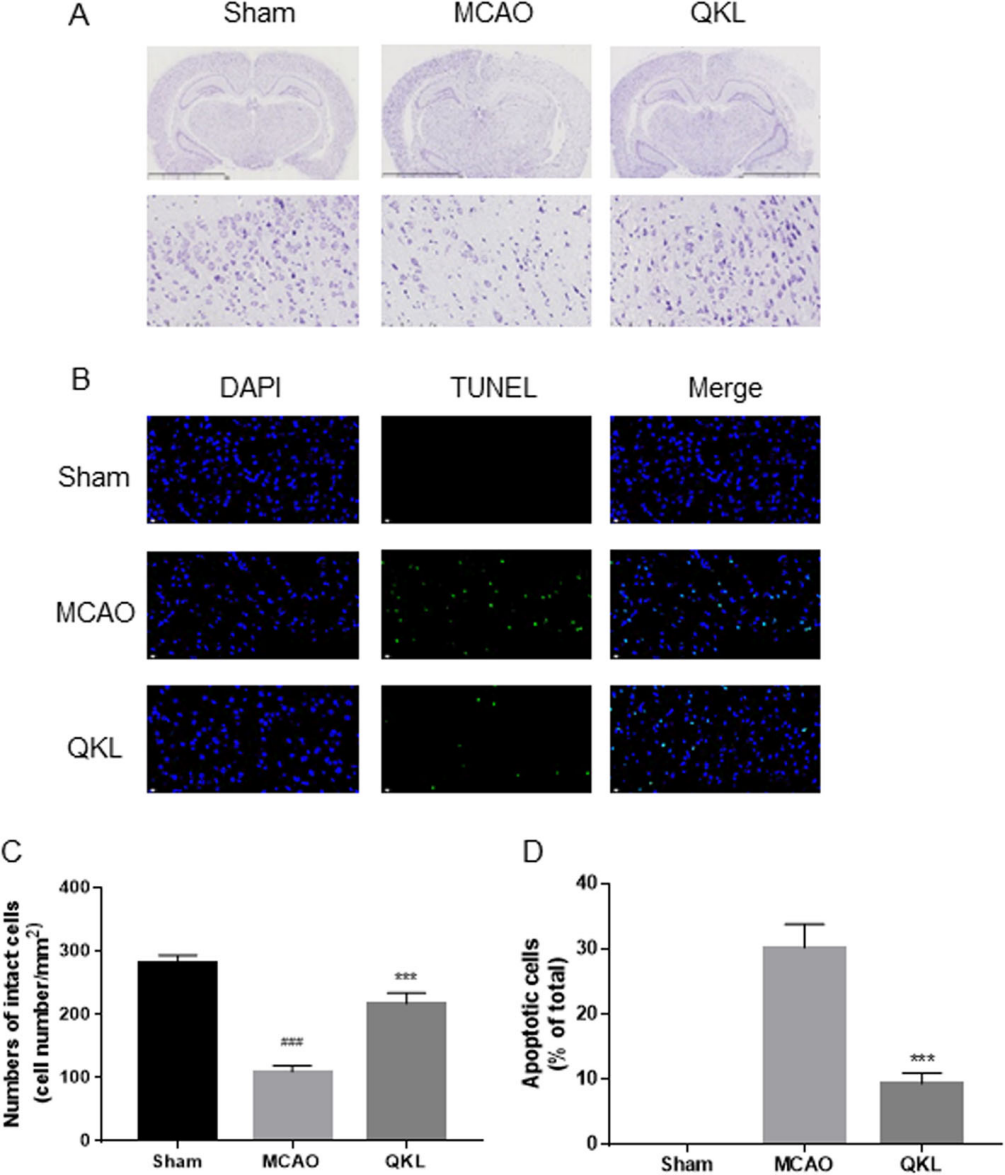
Anti-apoptosis effects of QKL. **a** Representative pictures of Nissl staining and **b** representative images of cell apoptosis stained with the TUNEL method. **c** Relevant quantitative analysis of intact cell number and **d** rate of apoptotic cells in each group. Data points indicate means ± SEM. Vs Sham group, ### < 0.001, Vs MCAO group, *** *p* < 0.001. At least three independent experiments were performed for each group

Cell damage after cerebral ischemia-reperfusion injury was estimated using Nissl staining. As shown in Fig. 3a-b, most cells shrank in size, although the intercellular space was increased and had deep colour staining in the MCAO group. However, these characteristic changes were improved after QKL treatment. Furthermore, more intact cells were present in the penumbra of the ischemic cortex in the QKL-treated rodents than in the MCAO group (Vs MCAO, *p* = 0.0003). Similarly, terminal deoxynucleotidyl transferase dUTP nick end labelling (TUNEL) positive cells were examined in the cortex after MCAO. As shown in Fig. 3c-d, QKL reduced the percentage of apoptotic cells significantly (Vs MCAO, *p* = 0.0006).

### Effects of QKL on upregulation of inflammatory cytokines by cerebral ischemia

**Fig. 4:**
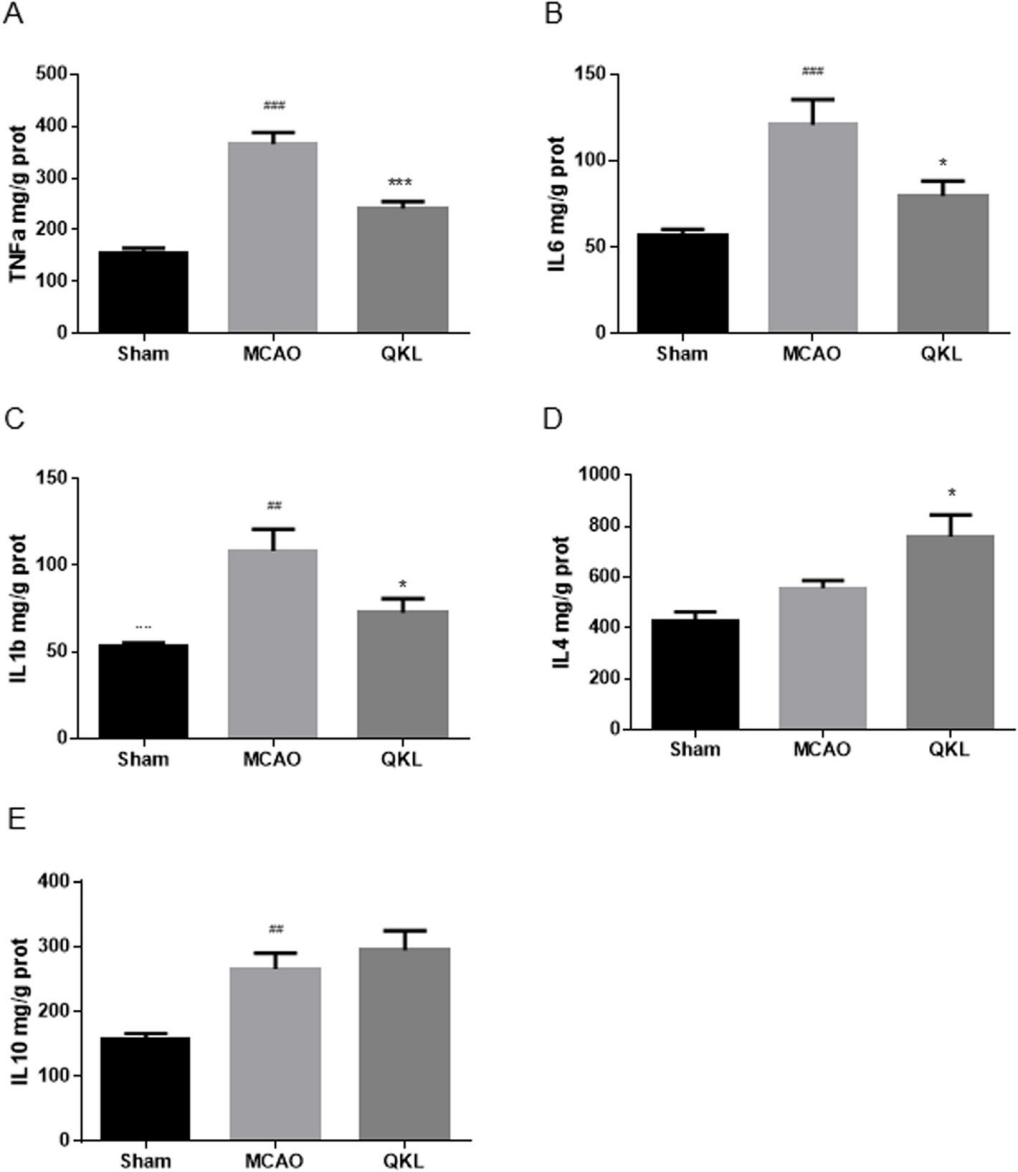
Analysis of the expression of pro-inflammatory cytokines, TNF-α, IL-6 and IL-1β, and anti-inflammatory cytokines, IL-4 and IL-10. **a**-**c** analysis of pro-inflammatory cytokines using enzyme-linked immunosorbent assay (ELISA) method for TNF-α, IL-6 and IL-1β, respectively. **d**-**e** analysis of anti-inflammatory cytokines using the ELISA method for IL-4 and IL-10, respectively. Data points indicate means ± SEM. Vs Sham group, ## < 0.01, ### < 0.001, Vs MCAO group, * *p* < 0.05, *** *p* < 0.001. At least three independent experiments were performed for each group

Enzyme linked immunosorbent assay (ELISA) analysis was used to evaluate the amount of pro-inflammatory (TNF-α, IL-1β and IL-6) and anti-inflammatory cytokines (IL-4 and IL-10). As shown in Fig. 4a, QKL treatment decreased TNFα significantly (Vs MCAO, *p =* 0.0001), after TNF-α had been upregulated by the cerebral ischemia-reperfusion injury. A similar phenomenon was observed when detecting IL-6 and IL-1β (Vs MCAO, *p* = 0.02, *p* = 0.0227, respectively), which is shown in Fig. 4b-c. The anti-inflammatory factors were upregulated in the QKL-treatment group compared to the MCAO group. QKL significantly increased the level of IL-4 (Vs MCAO, *p* = 0.0419) and increased the level of IL-10, but this difference was not significant (Vs MCAO, *p* > 0.05).

### Effects of QKL on the AMPK/NLRP3 inflammasome signalling pathway

**Fig. 5:**
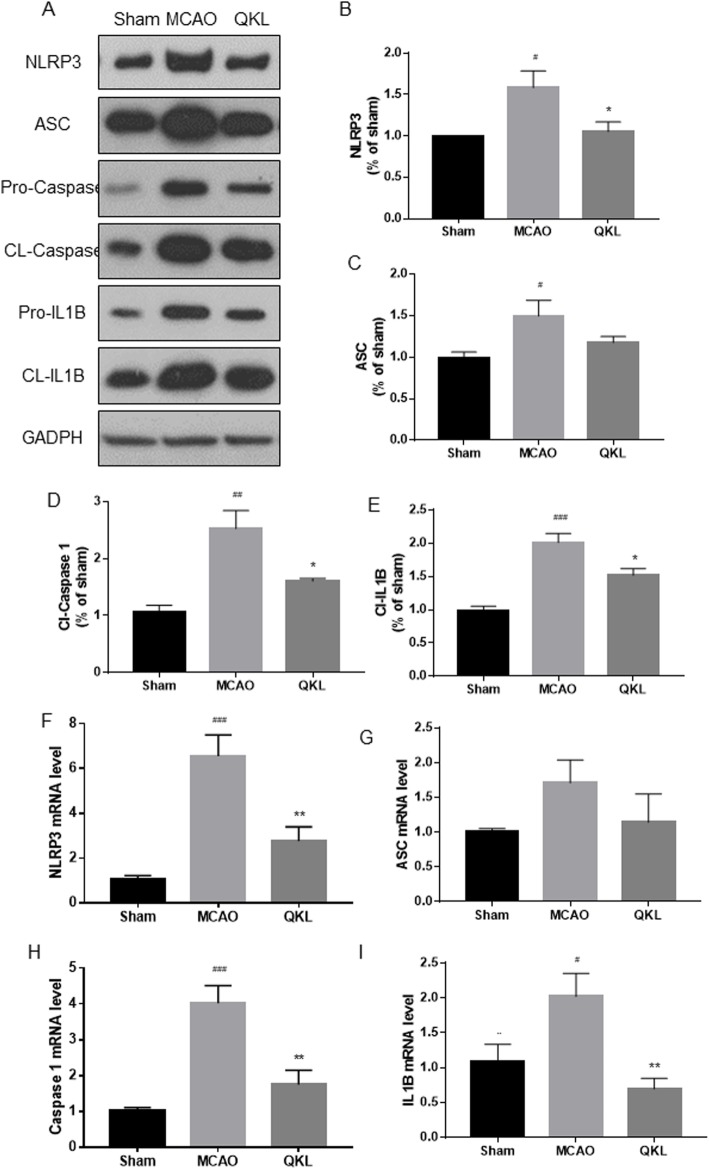
QKL inhibited NLRP3 inflammasome activation post-stroke. (A) Western blot analysis of NLRP3, ASC, pro-caspase 1, cleaved-caspase 1, pro-IL-1β, cleaved-IL-1β and GAPDH. (B-E) Quantitative analysis of NLRP3, ASC, Cl-caspase 1 and cl-IL-1β expression. (F-I) Quantitative polymerase chain reaction (QPCR) analysis of NLRP3, ASC, caspase 1 and IL-1β mRNA. Data points indicate means ± SEM. Vs Sham group, # < 0.05, ## < 0.01, ### < 0.001, Vs MCAO group, * *p* < 0.05, ** *p* < 0.01. At least three independent experiments were performed for each group

**Fig. 6:**
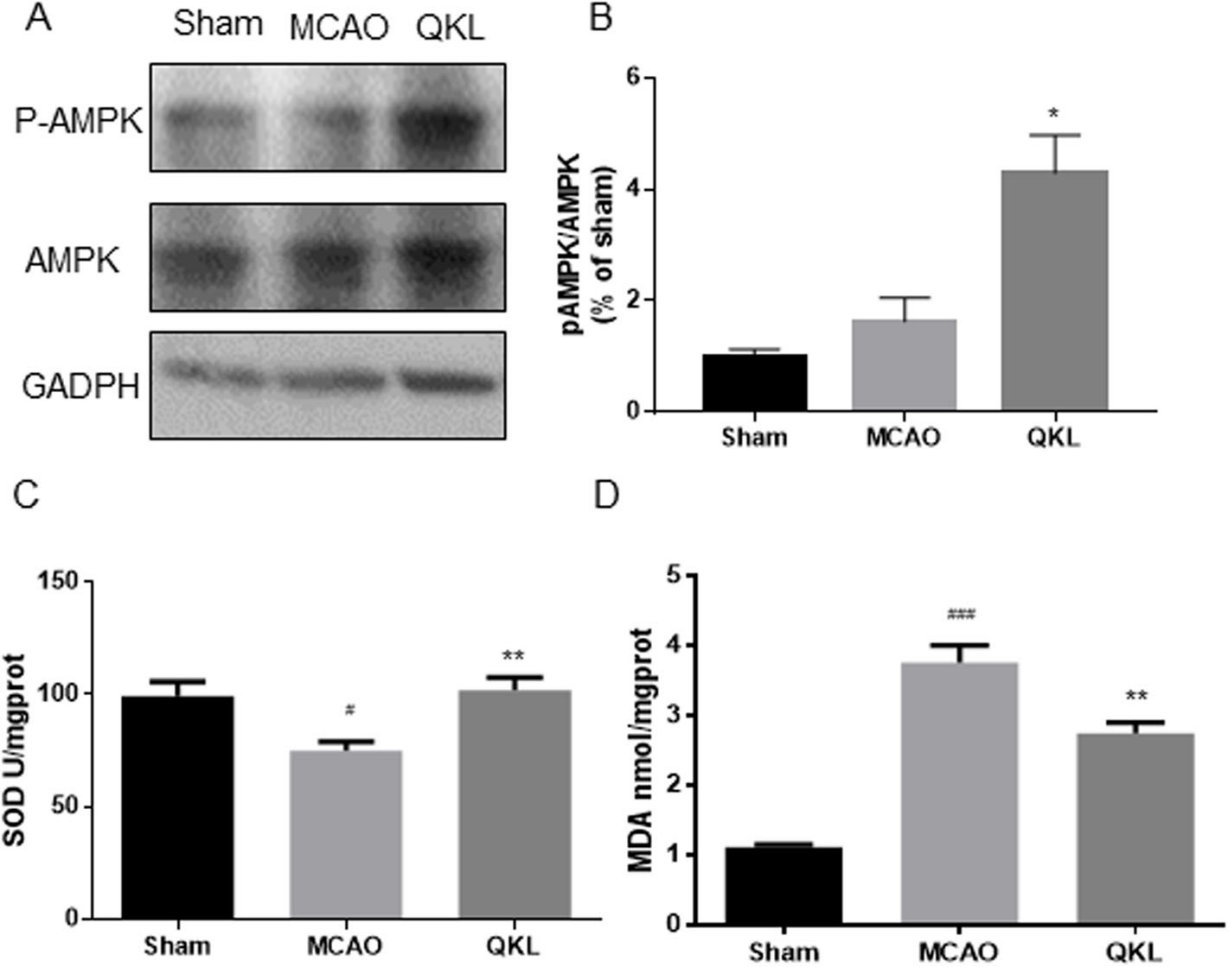
QKL activated AMPK and decreased oxidative stress levels. **a** Western blot analysis of p-AMPK, AMPK and GAPDH. **b** Quantitative analysis of the p-AMPK: AMPK expression ratio. **c** Effect of QKL on the SOD level. **d** Effect of QKL on the MDA level. Data points indicate means ± SEM. Vs Sham group, # < 0.05, ### < 0.001, Vs MCAO group, * *p* < 0.05, ** *p* < 0.01. At least three independent experiments were performed for each group

To understand the anti-stroke mechanism of QKL, we determined the protein expression levels of NLRP3, ASC, pro-caspase-1, cleaved-caspase-1, pro-IL-1β and cleaved-IL-1β in the brain tissue of rats (Fig. 5a). As shown in Fig. 5b-e, the level of NLRP3, ASC, Cl-caspase-1, and Cl-IL-1β were increased in the MCAO group compared to the Sham group (Vs Sham, *p* = 0.023, *p* = 0.0330, *p* = 0.0010 and *p* = 0.0001, respectively). The levels of NLRP3, Cl-caspase-1, and Cl-IL-1β were significantly decreased after QKL treatment (Vs MCAO, *p* = 0.0371, *p* = 0.0165, *p* = 0.0157, respectively). The level of ASC was also increased after QKL treatment even though a significant difference was not observed (Vs MCAO, *p* = 0.1816). We used quantitative polymerase chain reaction (qPCR) to further evaluate the mRNA levels of NLRP3, ASC, caspase 1, and IL-1β. As shown in Fig. 5f-i, the level of NLRP3, Cl-caspase-1, and IL-1β mRNA were significantly reduced after QKL treatment (Vs MCAO, *p* = 0.0031, *p* = 0.0017, *p* = 0.0057, respectively) and the level of ASC mRNA was reduced without significance (Vs MCAO, *p* = 0.3437), which corresponded to the Western blot analysis result. We further investigated the activation of AMPK, a recently identified upstream protein of the NLRP3 inflammasome. The results indicated that QKL treatment activated AMPK via phosphorylation (Vs MCAO, *p* = 0.0125) as shown in Fig. 6a-b.

### Effects of QKL on the oxidative stress

An over accumulation of free radicals induced by cerebral ischemia-reperfusion injury can activate the NLRP3 inflammasome. SOD is an effective free-radical scavenger and an important antioxidant enzyme that scavenges oxygen free radicals after cerebral ischemic events. In Fig. 6c, our data show that QKL increased the level of SOD (F (2, 15) = 7.702, *p* = 0.005), which reduced the oxidative stress (Vs MCAO, *p* = 0.0053). The level of MDA, an important lipid-peroxidation product, was also found significant different among these groups (F (2, 15) = 4.813, *p* = 0.0001). In the ischemic brain, MDA was found to be downregulated after QKL treatment (Vs MCAO, *p* = 0.0015), as shown in Fig. 6d.

## Discussion

Cerebral ischemia diminishes the oxygen supply, which damages the brain cells leading to neurological dysfunction and cerebral infarction. Challenges still exist in the management of cerebral ischemia, and more efficient agents to treat cerebral ischemia have yet to be identified. QKL is a famous CFDA approved Chinese medicine that has been clinically used throughout China for over 30 years. Understanding of biological mechanism of QKL from experimental studies may help developing potential clinical strategies of QKL to treat cerebral ischemia. The present study showed that QKL injection protected the brain against cerebral ischemia-reperfusion injury, including improved rCBF, decreased neurological deficits and brain infarct volumes, inhibited BBB injuries and suppressed post-stroke inflammatory responses. Using Nissl staining and TUNEL analysis, we found that QKL suppressed brain cell apoptosis, which is the biological mechanism leading to the clinical symptoms of cerebral ischemia. In line with our study, other studies indicated that QKL injection improves neurological function, suppresses BBB dysfunction, and inhibits inflammatory responses [19, 25]. However, the underlying mechanism of the QKL anti-ischemic effect remains unknown. Baicalin, geniposide, cholic acid, hyodeoxycholic acid, chlorogenic acid and neochlorogenic acid were identified as six dominant ingredients absorbed into blood according to UPLC-MS/MS analysis [31]. These ingredients were reported to protect brain against cerebral ischemia.

Recent studies highlighted anti-inflammation effect of QKL injection. Our results indicated that QKL was able to modulate the NLRP3 inflammasome activation. Indeed, no matter whether the target is upstream or downstream in the NLRP3 inflammasome pathway at the molecular level, modulating the expression, assembly, activation and secretion of the NLRP3 inflammasome and IL-1β exerts a protective effect on cerebral ischemia-reperfusion injury. Better functional outcomes, decreased infarction volumes and oedema formations, preservation of BBB permeability, and reduced inflammatory pathology against cerebral ischemia-reperfusion injury was observed in a preclinical study [32]. QKL treatment inhibited the transcription of NLRP3, caspase 1, and IL-1β, which suppressed activation of NLRP3 inflammasome signalling pathway. Western blot analysis also indicated that expression of the NLRP3 protein was downregulated by QKL treatment. NLRP3 recruited ASC, which in turn recruited caspase-1, causing its activation. Cellular pyroptosis was induced by activated caspase-1. And caspase-1 also processed pro-IL-1β to a mature form, which was rapidly secreted into the extracellular matrix activating inflammatory responses. Therefore, activated caspase-1 and IL-1β are two important molecules to evaluate the activation of the NLRP3 inflammasome. Our results showed that QKL reduced the level of Cl-caspase-1 and Cl-IL-1β, which is to say that QKL was able to influence NLRP3 inflammasome activation through related protein expressions and functions of both molecules.

To further understand the underlying mechanism behind QKL in NLRP3 inflammasome activation, we tested AMPK activation, which was considered to be a target for modulating inflammation and oxidative stress in multiple pathophysiologic conditions [33, 34]. QKL treatment activated the phosphorylation of AMPK, improved the level of SOD, and decreased the level of MDA, indicating that QKL plays a role in AMPK signalling and oxidative stress. As mentioned previously, activation of the AMPK signal inhibits the NLRP3 inflammasome via multiple biological mechanisms. Blocking AMPK activation induced by molecular inhibitors, such as compound C or sunitinib, was associated with an over-active NLRP3 inflammasome signalling pathway in vivo and in vitro [35, 36]. The direct interaction between AMPK and NLRP3 protein has not been reported to date. The most discussed mechanism is activated AMPK signal regulation of mitochondrial homeostasis, which is strongly connected to NLRP3 activation. Following mitochondrial dysfunction, AMPK is phosphorylated and activated to avoid risks of bioenergetic deficiencies. Furthermore, mitochondria are involved in many pathological processes as a consequence of their central role in reactive oxygen species (ROS) production [37]. AMPK is likely to be involved in cellular defenses against oxidative stress induced by mitochondrial ROS through increased SOD levels [38]. Previous research has indicated that high levels of ROS under various cellular stresses will activate the NLRP3 inflammasome signal pathway [39]. In detail, excess ROS induces the ROS scavenging protein thioredoxin from thioredoxin interacting/inhibiting protein (TXNIP), which binds to NLRP3 protein and modulates its assembly via oligomerization [40]. Revealing the functional interaction between AMPK and NLRP3 is important, as it demonstrates a novel relationship between inflammation/ROS and energy homeostasis.

The present research was the first preclinical study to examine the regulatory effect of QKL injection on AMPK/NLRP3 inflammasome pathway activation following cerebral ischemia-reperfusion injury. Actually, some ingredients in QKL injection have been considered to be critical regulators in the activation of AMPK/NLRP3 inflammasome pathways, such as isoliquiritigenin [41], cholic acid [42] and baicalin [43, 44]. However, the direct interaction between the ingredients and the core protein in the AMPK/NLRP3 pathway remained unknown. Future studies should seek to identify the potential ingredients in the QKL injection that target the AMPK/NLRP3 inflammasome signal pathway in the treatment of cerebral ischemia. The identification of the specific ingredient will facilitate the translation of basic science to the clinical setting.

## Conclusions

In conclusion, this study elucidated that QKL relieved cerebral ischemia reperfusion injury and suppressed inflammatory response, at least in part, by inhibiting AMPK-mediated the activation of NLRP3 inflammasome. Therefore, our data might supply a new insight into the potential of QKL injection in the treatment of cerebral ischemia and help to understand the role of QKL injection on immune system in the brain.

## Data Availability

The supporting materials can be obtained upon request via email to the corresponding author.

## Abbreviations

AMPK: AMP-activated protein kinase
ASC: Apoptosis-associated speck-like protein containing a CARD
BBB: Blood brain barrier
CNS: Central nervous system
DAMPs: Damage-associated molecular patterns
NLRP3: Nucleotide-binding oligomerization domain (NOD)-like receptor (NLR) Pyrin domain containing 3
PAMPs: Pathogen-associated molecular patterns
PRRs: Pattern recognition receptors
QKL: Qingkailing
tMCAO: Transient MCAO
